# Health behaviors, obesity, and marital status among cancer survivors: a MEPS study

**DOI:** 10.1007/s11764-022-01269-x

**Published:** 2022-11-21

**Authors:** Lixin Song, Ting Guan, Peiran Guo, Xianming Tan, Ashley Leak Bryant, William A. Wood, Anthony D. Sung, Erin Elizabeth Kent, Thomas C. Keyserling

**Affiliations:** 1 School of Nursing, University of Texas Health Science Center at San Antonio (UTHSCSA), 7703 Floyd Curl Drive, Mail Code 7947, San Antonio, TX 78229 USA; 2 Mays Cancer Center, University of Texas Health Science Center at San Antonio, San Antonio, TX 78229 USA; 3 School of Social Work in the David B. Falk College of Sport and Human Dynamics, University of Syracuse, Syracuse, NY 13244 USA; 4 School of Nursing, University of North Carolina at Chapel Hill (UNC-CH), Chapel Hill, NC 27599 USA; 5 Lineberger Comprehensive Cancer Center, UNC-CH, Chapel Hill, NC 27599 USA; 6 Gillings School of Global Public Health, UNC-CH, Chapel Hill, NC 27599 USA; 7 Department of Medicine School of Medicine, UNC-CH, Chapel Hill, NC 27599 USA; 8 Department of Medicine School of Medicine, Duke University, Durham, NC 27710 USA

**Keywords:** Cancer, Survivorship, Medical Expenditure Panel Survey (MEPS), Marital status, Health behavior, Weighted multivariate analysis

## Abstract

**Purpose:**

Promoting positive health behaviors helps improve cancer survivors’ health outcomes during survivorship; however, little is known about whether health behaviors differ by marital status. The purpose is to examine whether health behaviors and obesity among cancer survivors vary by marital status and whether the type of cancer and sociodemographic factors influence the relationship.

**Methods:**

We examined smoking, physical activity, and body mass index (BMI) among 1880 individuals diagnosed with prostate, breast, or colon cancer who were identified from the 2011–2017 Medical Expenditure Panel Survey (MEPS). We used Rao-Scott design-adjusted chi-square tests and weighted multivariable logistic regressions to achieve the research aims.

**Results:**

Current smoking behavior and BMI were significantly related to marital status. Survivors who had never married were the most likely to be current smokers across all cancer types. Married survivors were the most likely to be overweight or obese, while widowed survivors were the most likely to have a normal weight. The relationship between BMI and marital status varied by cancer type. Widowed colon cancer survivors were least likely to be overweight or obese; divorced/separated colon cancer survivors were most likely to be obese or overweight. Health behavior disparities were found among cancer survivors of different age, sex, race, and levels of education and income.

**Conclusions:**

There were relationships between marital status, health behaviors, and obesity among cancer survivors.

**Implications for Cancer Survivors:**

Our results suggested that relationship status and sociodemographic factors need to be considered in tailoring interventions to promote health behaviors among cancer survivors.

**Supplementary information:**

The online version contains supplementary material available at 10.1007/s11764-022-01269-x.

## Introduction

Health-promoting behaviors benefit cardiovascular wellbeing [1], prevent and manage cancer- and treatment-related side effects and complications [2], and prevent recurring cancer [3]. The American Cancer Society (ACS) and the National Comprehensive Cancer Network (NCCN) recommend improving cancer survivors’ health outcomes by promoting healthy behaviors during survivorship [4, 5]. Specifically, cancer survivors should engage in regular physical activity, maintain a healthy weight, achieve a healthy dietary pattern, and not smoke [4]. Overall adherence to these recommendations has been disappointing among cancer survivors [6]: 9–10% of cancer survivors are current smokers, less than half meet the physical activity recommendation [7], and about 30% are obese [8].

Social networks and social support are related to health behavior change [9], which can ultimately influence health outcomes [10]. Marriage can provide social support that influences an individual’s health behaviors and related outcomes. As Waite indicated, “Marriage provides individuals—especially men—with someone who monitors their health and health-related behaviors and who encourages self-regulation” [11]. Marriage may mobilize social and emotional support to encourage health-promoting behaviors [10] and can provide meaning, purpose, and obligation, which may inhibit risky behaviors and promote healthy behaviors [12]. Those who are married are more likely to be non-smokers [13], consume more vegetables [14], and participate in exercise more than singles [15]. However, husbands and wives (couples) are more likely to be obese than singles [16]. Methodologically, research has often grouped participants into married vs. not married or single; thus, researchers cannot differentiate the effects of divorced, widowed, separated, and never married on health behaviors and related outcomes. In the context of cancer, research has shown that cancer impacts the physical and psychological well-being of both the survivor and their family members, particularly their spouses [7]. However, how cancer survivors’ health behaviors are related to their marital status (i.e., married, divorced, widowed, separated, and never married) has rarely been examined.

Furthermore, survivors manage cancer and related issues in a context—e.g., cancer types [17] and sociodemographic factors such as sex [17], age [18], race [19], education [20], and geographic location [21]—all of which influence health behaviors. However, little is known about whether these factors have the same influence on the health behaviors of cancer survivors who have different marital statuses. To fill these gaps, this study aimed to examine whether cancer survivors’ health behaviors varied by marital status and whether the health behaviors-marital status relationship differed by the cancer type and sociodemographic factors. Different from the general population, cancer survivorship creates a series of unique teachable moments for cancer survivors and their families when they frequently interact with healthcare professionals for treatment decision-making, symptom management, surveillance for cancer recurrence and second cancers, and management of their comorbid conditions [4, 5]. Knowledge generated from this study is critical for developing and implementing effective, targeted intervention programs to promote health behaviors among cancer survivors and their family members.

## Methods

### Study population

We analyzed the Medical Expenditure Panel Survey (MEPS) (https://www.meps.ahrq.gov/mepsweb/) data between 2011 and 2017. MEPS is a set of large-scale questionnaires administered to noninstitutionalized populations in the USA. Yabroff et al. have reported detailed information on MEPS methodology previously [22]. The overall response rates for MEPS 2011–2017 ranged from 44.2 to 56.3% [23]. We identified individuals as adult cancer survivors if they were ≥ 18 years old and responded “yes” to the question, “Have you ever been told by a doctor or other health professional that you had cancer or a malignancy of any kind?” Respondents were included in the analysis if they were diagnosed with prostate, breast, or colon cancer—three of the most common types of solid tumors in the USA [24].

### Measurement

Health behaviors assessed included smoking and physical activity. We obtained information on these behaviors based on a “yes” response to the survey items “currently smoke” and “currently spend half hour or more in moderate to vigorous physical activity (e.g., brisk walking, dancing, running, and fast swimming) at least five times a week” [25]. Obesity status was assessed according to BMI cutpoints outlined by the Centers for Disease Control and Prevention [26]: underweight (BMI < 18.5), normal (BMI = 18.5–24.9), overweight (BMI = 25.0–29.9), and obese (BMI ≥ 30.0) (Note: cancer survivors who were underweight were 1.12% of the respondents, and thus, excluded from the analysis due to a small sample size).

Marital status was categorized in the survey data as married, widowed, divorced/separated, and never married. We obtained survivors’ demographic characteristics from the MEPS household component, including age, sex, race/ethnicity, education, family income, and insurance coverage [27]. We categorized race (White vs non-White), education (< high school degree or General Educational Development (GED), high school or GED, and > high school or GED), and insurance status (private, public, uninsured). Based on the federal poverty guideline, we classified family income into five categories: poor (< 100%); near poor (100– < 125%); low (125– < 200%); middle (200– < 400%); and high (≥ 400%) income. Data about the time from cancer diagnosis, cancer status, and treatment status are not available in the survey thus, are not included in the analyses.

### Data analysis

Using the Rao-Scott design-adjusted chi-square test—a design-adjusted version of the Pearson chi-square test [28], we calculated weighted percentages (SAS Proc surveyfreq) of survivor health behaviors and compared health behaviors among cancer survivors with different marital status. To examine whether the relationship between health behaviors and marital status differed by cancer type, we conducted weighted multivariable logistic regressions (SAS Proc surveylogistic) and modeled cancer type, marital status, and the cancer type*marital status interaction on health behaviors. We used a weighted regression approach with personal weights to account for MEPS’ design complexity. To examine whether sociodemographic factors influenced the relationship between health behaviors and marital status, we added survivors’ characteristics into the models obtained in the previous steps. We performed stepwise variable selection to obtain parsimonious models.

## Results

We identified 1880 individuals who reported being diagnosed with breast (50.8%), prostate (35.0%), and colon (14.2%) cancers. The mean age was 68.7 and a majority were female (57.4%); were White (84.4%); had a greater than high school degree or GED education (57.7%); were middle or high income (25.5% and 44.8%, respectively); and had private health insurance (64.0%). These cancer survivors reported their marital status as married (59.3%), widowed (19.3%), divorced/separated (16.0%), and never married (5.5%) (Table 1).

**Table 1:** Sample characteristics

Characteristics	*N*	%
Sex		
Female	1075	57.38%
Male	805	42.62%
Race		
White	1336	84.38%
Non-White^a^	542	15.62%
Hispanic		
Yes	244	6.07%
Poverty status^b^		
Poor	283	11.47%
Near poor	102	4.67%
Low	301	13.49%
Middle	512	25.55%
High	682	44.82%
Marital status		
Married	1022	59.28%
Widowed	392	19.25%
Divorced/separated	339	15.97%
Never married	127	5.50%
Education		
≤ 12th grade	332	11.50%
GED or high school degree	574	30.79%
> high school degree	957	57.71%
Insurance coverage		
Private	1088	64.02%
Public^c^	761	34.71%
Uninsured	31	1.27%
Census region		
Northeast	338	18.33%
Midwest	395	22.64%
South	725	37.83%
West	422	21.20%
Cancer type		
Breast	931	50.80%
Prostate	654	34.96%
Colon	295	14.24%
	Mean	SD
Age	68.72	0.36

^a^Non-White included Black, American Indian/Alaska Native, Asian, Native Hawaiian/Pacific Islander, and multiple races

^b^Family income was classified into five poverty categories: poor (< 100%), near poor (100– < 125%), low (125– < 200%), middle (200– < 400%), and high income (≥ 400%)

^c^Persons identified as covered by public insurance are those reporting coverage under TRICARE, Medicare, Medicaid or SCHIP, or other public hospital/physician programs

### Health behaviors and marital status

Approximately 18.1% of survivors were current smokers, and 41.4% reported currently engaging in half an hour or more of moderate to vigorous physical activity at least five times a week. Approximately 32.6% and 37.0% of the survivors were overweight or obese, respectively. Current non-smoking behavior was significantly related to marital status (*p* < 0.0001). Widowed survivors were the least likely to be a current smoker, while never married were the most likely to currently smoke. Obesity was significantly related to marital status (*p* < 0.01): married survivors were the most likely to be overweight or obese, whereas widowed survivors were the most likely to have a normal weight (Table 2).

**Table 2:** The association between health behaviors and marital status

Health behaviors	Total		Married		Widowed		Divorced/separated		Never married		*p* value^c^
	***N***	**%**	***N***	**%**	***N***	**%**	***N***	**%**	***N***	**%**	
Currently non-smoke (yes/no)	1540	81.91%	860	93.42%	334	93.52%	253	85.94%	93	78.36%	< .0001
Physical activity (yes/no)	778	41.38%	449	46.65%	136	39.74%	145	44.97%	48	40.27%	0.188
BMI											
Normal	521	27.71%	288	30.92%	121	36.94%	79	24.51%	33	31.37%	0.0033
Overweight (BMI = 25.0–29.9)	613	32.61%	299	27.35%	123	29.47%	142	40.20%	49	28.17%	
Obese (BMI ≥ 30.0)	696	37.02%	411	40.63%	131	31.43%	112	34.45%	42	38.84%	

^c^The *p* values are based on Rao-Scott design-adjusted chi-square test to examine the association between health behaviors and marital status

### Health behaviors, marital status, and cancer type

The association between BMI and marital status differed by cancer type. Widowed colon cancer survivors had the lowest odds ratio (0.24) (95% CI [0.11–0.53]) of being obese or overweight, and divorced/separated colon cancer survivors had the highest odds ratio (1.18) (95% CI [0.60–2.31]) of being obese or overweight (Table 3).

**Table 3:** Health behaviors and marital status in the context of different cancer types

Health behaviors	Current non-smoker (Ref: current smoker)				Adequate physical activity (Ref: no physical activity)				Increased or high BMI (Ref: normal BMI)			
	*β*	SE	Odds ratio	95% CI	*β*	SE	Odds ratio	95% CI	*β*	SE	Odds ratio	95% CI
Marital status (ref: married)												
Divorced/separated	− 1.10	0.51	0.33*	0.12–0.90	− 0.74	0.38	0.48	0.22–1.01	0.17	0.34	1.18	0.60–2.31
Widowed	− 0.78	0.65	0.46	0.13–1.62	− 0.67	0.47	0.51	0.20–1.29	− 1.41	0.39	0.24***	0.11–0.53
Never married	− 0.62	0.85	0.54	0.10–2.88	− 0.58	0.6	0.56	0.17–1.81	− 0.39	0.51	0.68	0.25–1.84
Cancer type (ref: colon)												
Breast	0.49	0.45	1.64	0.68–3.96	− 0.25	0.26	0.78	0.47–1.28	− 0.54	0.23	0.58*	0.37–0.90
Prostate	0.28	0.45	1.33	0.54–3.24	− 0.11	0.24	0.89	0.56–1.42	− 0.35	0.22	0.70	0.46–1.08
Marital status * cancer type (ref: married * colon)												
Widowed*breast cancer	1.07	0.76	2.93	0.65–13.10	0.44	0.52	1.55	0.56–4.33	1.73	0.45	5.64***	2.34–13.63
Widowed*prostate cancer	1.04	0.87	2.84	0.52–15.57	0.57	0.59	1.77	0.56–5.60	1.14	0.48	3.14*	1.22–8.04
Divorced/separated*breast cancer	0.35	0.66	1.41	0.39–5.13	0.75	0.44	2.11	0.88–5.03	0.47	0.41	1.60	0.71–3.55
Divorced/separated*prostate cancer	0.29	0.64	1.33	0.38–4.65	0.88	0.48	2.42	0.95–6.17	0.21	0.46	1.23	0.50–3.01
Never married*breast cancer	− 1.13	0.97	0.32	0.05–2.15	0.37	0.72	1.45	0.35–6.00	0.35	0.6	1.43	0.44–4.61
Never married*prostate cancer	− 0.45	1.00	0.64	0.09–4.57	0.44	0.73	1.56	0.38–6.45	0.69	0.61	2.00	0.60–6.66

*p < .05; **p < .01; ***p < .001

### Health behaviors and marital status in the context of sociodemographic factors

**Fig. 1:**
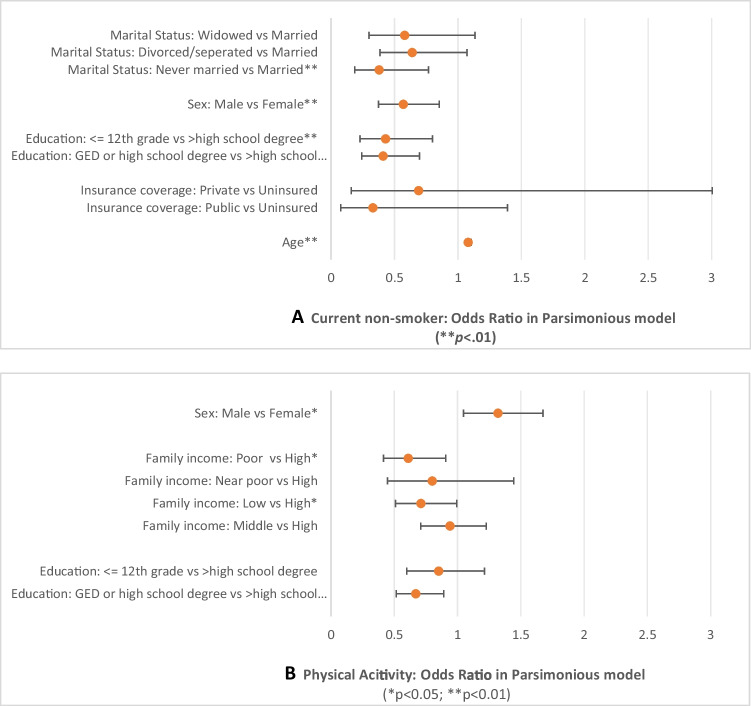
Influencing factors of smoking and physical activity behaviors

The results of the full and parsimonious models are displayed in the Online Appendix Table. In the parsimonious model that excluded the nonsignificant factors (Fig. 1A), the never married survivors had the highest odds ratio of being a smoker among all people with different marital status (*p* < 0.01), indicating never married survivors were the most likely to smoke currently. Current non-smoking behavior also differed by age, sex, and education. Older survivors were less likely to smoke currently (*p* < 0.01). Compared to females, males with cancer were more likely to smoke (*p* < 0.01). Survivors with a high school/GED or lower educational status were more likely to be current smokers as compared to survivors with more advanced education (both *ps* < 0.01).

Physical activity among survivors (Fig. 1B) differed by sex, education, and poverty status. Compared to male survivors, women were less likely to be physically active (*p* < 0.05). Survivors with a high school degree or GED were less likely to be active physically than those with a more than high school/GED education (*p* < 0.01). Survivors with low income were less likely to be physically active compared to those with high income (both *ps* < 0.05).

**Fig. 2:**
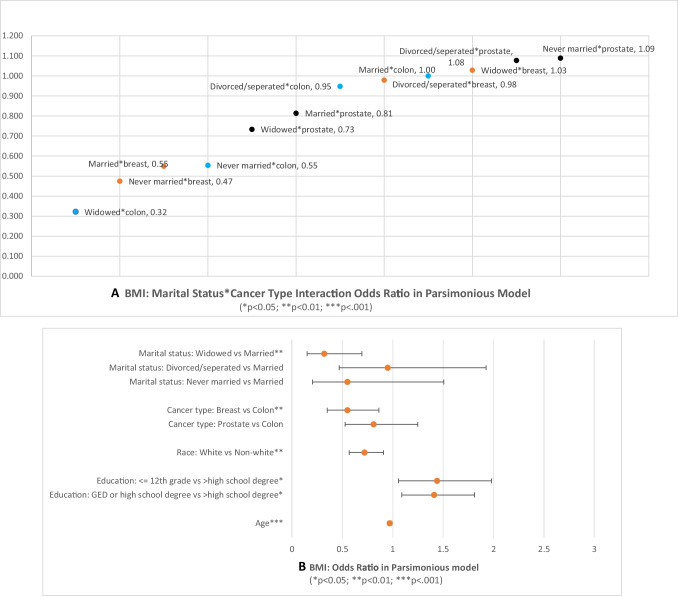
Influencing factors of BMI

The relationships between BMI and marital status continued to be significant after controlling for the effects of cancer type and sociodemographic factors (Fig. 2A). Among survivors with different types of cancer, widowed colon cancer survivors had the lowest odds ratio (0.32) (95% CI [0.15, 0.68]) of being obese or overweight, and never married prostate cancer survivors had the highest odds ratio (1.09) (95% CI [0.76–7.74]) of being obese or overweight. Figure 2B shows that BMI differed by age, race, and education. Older survivors were less likely to have a normal BMI (*p* < 0.001). Non-White survivors were more likely to be overweight and obese than their White counterparts (*p* < 0.01). Survivors with a high school/GED or lower educational status were more likely to be overweight or obese than those with a higher than high school/GED education (both *ps* < 0.05).

## Discussion

To our knowledge, this study using MEPS data is the first to examine how selected health promoting and adverse health behaviors are associated with marital status (i.e., married, widowed, divorced/separated, and never married) among survivors with different types of cancer in the context of their sociodemographic factors. Current smoking behavior and BMI (a proxy for obesity) were related to marital status. Among patients with prostate, breast, and colon cancer, those who were never married had higher rates of smoking. Divorced/separated survivors were the most likely to be overweight, married survivors were the most likely to be obese, and those who were widowed were the most likely to have normal weight. The relationship between obesity and marital status varied by cancer type. We also identified disparities in health behaviors among cancer survivors by age, sex, race, education, and income. The results from this study inform development and implementation of tailored interventions to enhance healthy behaviors among cancer survivors with varying sociodemographic backgrounds.

Although cancer diagnosis and treatment offer survivors and their families the opportunity (e.g., education, skills training) to create healthy behavior change and promote positive outcomes, our findings indicate that some cancer survivors continue to engage in unhealthy behaviors. Approximately 18% of survivors of breast, colorectal, and prostate cancer in this MEPS study were current smokers as compared to 20.8% in the US general population [29]. Findings from other research that included survivors with breast, cervical, colorectal, and prostate cancer reported higher rates than our study [21]. About 41% of cancer survivors reported that they spend at least half an hour in moderate to vigorous physical activity more than five times a week, meeting the physical activity recommendation of ACS [4]. This finding is within the range reported by LeMasters et al. (30.3–46.6%) [17] but lower than that from a state-specific samples of randomly dialed telephone survey (~ 78%) using the Behavioral Risk Factor Surveillance System (BRFSS) [21]. The prevalence of overweight (32.61%) and obese (37.02%) survivors is similar to the general American adult population (overweight and obese > 70%) [26] but higher than previously reported estimates using data from the BRFSS [21] and the National Health Interview Survey (NHIS) [8]. Our findings suggest that smoking cessation, physical activity engagement, and, most importantly, weight loss remain challenging in cancer survivors. Researchers and healthcare providers can take better advantage of the teachable moments of cancer diagnosis, treatment, and follow-up visits to clearly communicate with cancer survivors about health behaviors and engage them in effective programs to promote positive outcomes.

We found that the never married survivors, regardless of cancer types, were most likely to smoke. This finding is different from that of the general population as recently noted in a Morbidity and Mortality Weekly Report [29], which showed that the current smoking rate was the highest among adults who were divorced, separated, or widowed, followed by adults who were single, never married, or not living with a partner; and the lowest among those who were married or living with a partner. The high prevalence of smoking among never married cancer survivors may be related to a lack of influence, support, and social control over risky behaviors from spouses. Marriage can influence health behaviors directly through sanctioning or impeding and indirectly through internalizing norms about the behaviors [30]. Furthermore, compared to unpartnered people, adults who had a non-smoking partner or whose partners quit smoking were more likely to quit smoking [31]. These findings emphasize that never-married cancer survivors may need additional support to quit smoking.

We also found that current smoking status differed by survivors’ age, sex, and educational attainment. Specifically, those who were male, younger, and whose educational status was lower than high school were more likely to smoke. Findings about the relationships between smoking status, age, and gender have been mixed; however, the inverse association between smoking prevalence and educational attainment has been consistent. A review of studies of the general public reported that men used tobacco products at higher rates than women, and the significant gender differences in smoking are prevalent among younger adults but absent among older smokers [32]. In contrast, an earlier study of US cancer survivors using NHIS data found that smoking status differed significantly by age and age at diagnosis among men and women and that females had higher rates of being a current smoker than males, particularly among those 40 years of age or younger [8]. Social, economic, personal, and political influences all impact smoking prevalence and cessation [33]. For example, a smoker’s age is related to the stage of the smoking cessation process, and thus, smoking cessation programs might be improved by matching intervention strategies to a smoker’s age and their stage of readiness [34]. Our findings indicate that, although being diagnosed with cancer may motivate people to quit smoking, many factors can impede smoking cessation, including social and personal factors. Our results may contribute to development of tailored smoking cessation interventions based on cancer survivors’ marital status and their sociodemographic backgrounds. Cancer survivors who are male, younger, and have less education may benefit from smoking cessation strategies that meet their unique needs.

We also found that obesity was related to marital status, even after considering the effects of sociodemographic factors. Married survivors were the most likely to be obese, while widowed survivors were the most likely to have a normal weight. Among all cancer survivors with different marital status and types of cancer, widowed colon cancer survivors were least likely to be obese or overweight, and divorced/separated colon cancer survivors were most likely to be obese or overweight. This finding is consistent with research on the general population [35]. Marriage may provide role obligations for eating regular meals [36]. Conversely, a spouse’s death is a stressful life event that may cause loss of appetite and regular meals. A lack of support, influence, and social control over risky behaviors (e.g., overeating) from spouses may be related to overweight and obesity among divorced/separated colorectal survivors [30]. Our findings highlight the need for interventions that encourage both cancer survivors and their spouses to establish healthy eating patterns and reduce obesity and for tailored interventions to target survivors with different types of cancer and with different marital status, especially among colon cancer patients.

Although the association between physical activity and marital status was non-significant, our findings show that physical activity differed by survivors’ educational attainment and family income. Specifically, survivors with high school or lower education and those with poor and low family income were more likely to be physically inactive. Our finding is congruent with the results from an ACS’ study (*n* = 1160) of patients with breast, colorectal, and prostate cancer, which also found that physically inactive survivors were more likely to have lower education (≤ high school) and household income [37]. This disparity in physical activity by educational status has been well documented [38]. High levels of education provide individuals with increased knowledge of the benefits of physical activity, greater access to resources, and healthier influences from their social networks, which all facilitate physical activity [39]. Similarly, individuals who have higher incomes have more resources and locations to exercise, which facilitates physical activity [40]. Effective interventions are needed to increase physical activity for cancer survivors with low socioeconomic status.

The limitations of our study are as follows. First, we used the marital status reported at one time point, making it impossible to assess marital status as a time-varying variable. We, therefore, could not investigate whether changes in marital status affected health behaviors. In the large-scale MEPS surveys of family and individuals, there are valid discrepancies in the case of persons who are married but not living with their spouse, separated but cohabitating, or unmarried partners living together (MEPS considers them as separate family units) [41], all of which largely limited our ability to tease out the information about how living arrangements are related to health behaviors among cancer survivors with different marital status. Our study using MEPS also has low percentage of never married survivors (5.5%), which may be caused by the fact that cancer survivors skew older, and thus, have low never married proportion. Next, as several studies have indicated [42, 43], publicly available MEPS data do not include the time of cancer diagnosis; therefore, we could not examine the association between time since diagnosis and health behaviors tested about a decade ago [17]. And also, we used respondents’ self-report data, which may reduce the precision of estimation of health behaviors. Finally, the generalizability of our findings may be reduced because most respondents had a greater than high school education, middle to high income, and private health insurance, which is higher compared to cancer survivors using the Behavioral Risk Factor Surveillance System dataset [44].

Nonetheless, this study using MEPS extends the current research on the sociodemographic findings related to health behaviors in the general population to provide insight into how health behaviors are related to marital status among cancer survivors. Our findings suggest that interventions need to be tailored based on survivors’ marital status and sociodemographic characteristics in order to effectively promote healthy behaviors and to improve survivorship outcomes. Future research must develop and evaluate feasible supportive care interventions, especially for never-married cancer survivors to quit smoking and couple-based interventions for married survivors to reduce their weight and obesity. In addition, given that marital status is associated with smoking and BMI for cancer survivors, future research needs to address mechanisms that account for these associations, e.g., using dyadic analysis to understand the interpersonal influences and outcomes regarding health behaviors. Additionally, while this study grouped survivors based on their answer to a simple question of marital status, both married and unmarried survivors could have good or poor social support, living arrangement (living together or separately), and different time since cancer diagnosis. Future research must collect more comprehensive data to verify our study findings as well as to examine each of the subgroups—i.e., married vs unmarried with good vs poor support, living arrangement, and time since diagnosis —to further understand how social support influences health behaviors of individuals who have different marital status, living circumstance during the continuum of cancer survivorship. Future research should also include survivors who are partnered (vs married) so that the findings are more generalizable. Research using marital status as a time-varying variable may also help extrapolate how behaviors change over time. Lastly, the majority of the sample were White (84.4%), and future studies should focus on recruiting and retaining people of color to investigate how marital status impacts health behaviors. As social determinants play a critical role in shaping health behaviors, research is also needed to identify strategies to promote health behaviors among disadvantaged cancer survivors (i.e., rural communities, LGBTQIA).

## Conclusions

We used the MEPS data to examine relationships between health behaviors, sociodemographic factors, and marital status among cancer survivors with prostate, breast, and colon cancers. Our findings suggest that relationship status and sociodemographic factors need to be considered in tailoring interventions to improve health behaviors among cancer survivors.

## Supplementary information

Below is the link to the electronic supplementary material.

Supplementary file1 (DOCX 28.6 KB)
