# Supplementary material for: Health behaviors, obesity, and marital status among cancer survivors: a MEPS study
Source: J Cancer Surviv. 2022 Nov 21;17(2):499–508. doi: 10.1007/s11764-022-01269-x (PMC10036458; doi:10.1007/s11764-022-01269-x)
Supplement: Supplementary file 1 — Supplementary file1 (DOCX 28.6 KB) [file 11764_2022_1269_MOESM1_ESM.docx]

| **Appendix Table. The association between health behaviors and sociodemographic factors** | | | | | | | | | | | | | | | | | | | | |
| --- | --- | --- | --- | --- | --- | --- | --- | --- | --- | --- | --- | --- | --- | --- | --- | --- | --- | --- | --- | --- |
| **Health behaviors** | **Current non-smoker** | | | | | | **Adequate Physical Activity** | | | | | | | **Increased or High BMI** | | | | | |  |
|  | **Full model** | | | **Parsimonious model** | | | **Full model** | | | | **Parsimonious model** | | | **Full model** | | | **Parsimonious model** | | |  |
|  | **β** | **SE** | **OR** | **β** | **SE** | **OR** | **β** | **SE** | **Odds Ratio** | **β** | | **SE** | **OR** | **β** | **SE** | **OR** | **β** | **SE** | **OR** |  |
| **Marital status (ref: married)** |  |  |  |  |  |  |  |  |  |  | |  |  |  |  |  |  |  |  |  |
| Widowed | -1.54 | 0.71 | 0.21* | -0.54 | 0.34 | 0.58 | -0.32 | 0.47 | 0.73 |  | |  |  | -1.20 | 0.40 | 0.3** | -1.13 | 0.39 | 0.32** |  |
| Divorced/separated | -0.22 | 0.54 | 0.81 | -0.44 | 0.26 | 0.64 | -0.54 | 0.38 | 0.58 |  | |  |  | -0.05 | 0.36 | 0.96 | -0.05 | 0.36 | 0.95 |  |
| Never married | 0.28 | 0.88 | 1.32 | -0.97 | 0.36 | 0.38** | -0.36 | 0.59 | 0.70 |  | |  |  | -0.66 | 0.53 | 0.52 | -0.59 | 0.51 | 0.55 |  |
| **Cancer type (ref: colon)** |  |  |  |  |  |  |  |  |  |  | |  |  |  |  |  |  |  |  |  |
| Breast | 0.31 | 0.49 | 1.36 |  |  |  | 0.23 | 0.30 | 1.26 |  | |  |  | -0.77 | 0.30 | 0.46* | -0.60 | 0.23 | 0.55** |  |
| Prostate | -0.09 | 0.61 | 0.91 |  |  |  | -0.43 | 0.29 | 0.65 |  | |  |  | -0.09 | 0.24 | 0.92 | -0.21 | 0.22 | 0.81 |  |
| **Marital status*cancer type (ref: married * colon)** | | |  |  |  |  |  |  |  |  | |  |  |  |  |  |  |  |  |  |
| Widowed*breast cancer | 1.32 | 0.79 | 3.74 |  |  |  | 0.27 | 0.53 | 1.32 |  | |  |  | 1.81 | 0.47 | 6.12*** | 1.76 | 0.45 | 5.81*** |  |
| Widowed*prostate cancer | 1.50 | 0.94 | 4.48 |  |  |  | 0.31 | 0.59 | 1.36 |  | |  |  | 1.09 | 0.50 | 2.98* | 1.03 | 0.48 | 2.80* |  |
| Divorced/separated*breast | -0.09 | 0.66 | 0.91 |  |  |  | 0.61 | 0.43 | 1.84 |  | |  |  | 0.62 | 0.44 | 1.86 | 0.63 | 0.43 | 1.88 |  |
| Divorced/separated*prostate | -0.10 | 0.69 | 0.90 |  |  |  | 0.70 | 0.49 | 2.02 |  | |  |  | 0.33 | 0.47 | 1.39 | 0.33 | 0.47 | 1.40 |  |
| Never married*breast cancer | -1.51 | 0.98 | 0.22 |  |  |  | 0.29 | 0.70 | 1.34 |  | |  |  | 0.53 | 0.63 | 1.69 | 0.45 | 0.61 | 1.56 |  |
| Never married*prostate | -0.77 | 1.05 | 0.47 |  |  |  | 0.35 | 0.73 | 1.42 |  | |  |  | 0.96 | 0.61 | 2.62 | 0.88 | 0.59 | 2.42 |  |
| **Sex (ref: female)** |  |  |  |  |  |  |  |  |  |  | |  |  |  |  |  |  |  |  |  |
| Male | -0.33 | 0.45 | 0.72 | -0.57 | 0.21 | 0.57** | 0.83 | 0.31 | 2.30** | 0.28 | | 0.12 | 1.32* | -0.30 | 0.30 | 0.74 |  |  |  |  |
| **Race (ref: non-white)** |  |  |  |  |  |  |  |  |  |  | |  |  |  |  |  |  |  |  |  |
| White | 0.03 | 0.23 | 1.03 |  |  |  | 0.18 | 0.14 | 1.20 |  | |  |  | -0.31 | 0.12 | 0.73* | -0.33 | 0.12 | 0.72** |  |
| **Family income (ref: high)** |  |  |  |  |  |  |  |  |  |  | |  |  |  |  |  |  |  |  |  |
| Poor | -0.81 | 0.34 | 0.44* |  |  |  | -0.45 | 0.23 | 0.64* | -0.49 | | 0.2 | 0.61* | -0.07 | 0.19 | 0.93 |  |  |  |  |
| Near poor | -0.55 | 0.52 | 0.58 |  |  |  | -0.19 | 0.31 | 0.83 | -0.22 | | 0.3 | 0.80 | -0.11 | 0.25 | 0.9 |  |  |  |  |
| Low | -0.52 | 0.35 | 0.59 |  |  |  | -0.32 | 0.18 | 0.73 | -0.34 | | 0.17 | 0.71* | 0.23 | 0.2 | 1.26 |  |  |  |  |
| Middle | -0.34 | 0.35 | 0.71 |  |  |  | -0.05 | 0.15 | 0.95 | -0.07 | | 0.14 | 0.94 | 0.12 | 0.13 | 1.13 |  |  |  |  |
| **Education (ref: >high school degree)** | |  |  |  |  |  |  |  |  |  | |  |  |  |  |  |  |  |  |  |
| <= 12th grade | -0.66 | 0.36 | 0.52 | -0.85 | 0.32 | 0.43** | -0.13 | 0.17 | 0.88 | -0.16 | | 0.18 | 0.85 | 0.37 | 0.17 | 1.44* | 0.37 | 0.16 | 1.44* |  |
| GED or high school degree | -0.79 | 0.3 | 0.46** | -0.89 | 0.27 | 0.41** | -0.37 | 0.14 | 0.69** | -0.39 | | 0.14 | 0.67** | 0.33 | 0.13 | 1.39* | 0.34 | 0.13 | 1.41* |  |
| **Insurance (ref: uninsured)** | |  |  |  |  |  |  |  |  |  | |  |  |  |  |  |  |  |  |  |
| Private | -0.49 | 0.76 | 0.61 | -0.37 | 0.75 | 0.69 | 0.01 | 0.46 | 1.01 |  | |  |  | 0.06 | 0.52 | 1.06 |  |  |  |  |
| Public | -1.07 | 0.77 | 0.34 | -1.12 | 0.74 | 0.33 | 0.07 | 0.47 | 1.07 |  | |  |  | 0.02 | 0.53 | 1.02 |  |  |  |  |
| **Age** | 0.08 | 0.01 | 1.08*** | 0.08 | 0.01 | 1.08** | -0.01 | 0.01 | 0.99 |  | |  |  | -0.03 | 0.01 | 0.97*** | -0.03 | 0.01 | 0.97*** |  |
| *P ≤ .05. **P ≤ .01. ***P ≤ .001 | | | | | | | | | | | | | | | | | | | | |
